# Chapped Nipples

**Published:** 1843-04-15

**Authors:** 


Chapped Nipples.—Dr. Donné thinks that this troublesome and distressing complaint is frequently connected with an altered state of the milk itself. The milk will, in many such cases, be found to be poor and watery, not abundant, and containing more or less of mucous matters. The child being imperfectly fed, and finding difficulty in drawing the milk, pulls at the nipple more than usual; and perhaps, at the same time, its saliva is more than commonly saline, and thus increases the irritation.

